# Overexpression and Purification of *Gracilariopsis chorda* Carbonic Anhydrase (GcCAα3) in *Nicotiana benthamiana*, and Its Immobilization and Use in CO_2_ Hydration Reactions

**DOI:** 10.3389/fpls.2020.563721

**Published:** 2020-11-19

**Authors:** Md Abdur Razzak, Dong Wook Lee, Junho Lee, Inhwan Hwang

**Affiliations:** ^1^Division of Integrative Biosciences and Biotechnology, Pohang University of Science and Technology, Pohang, South Korea; ^2^Department of Bioenergy Science and Technology, Chonnam National University, Gwangju, South Korea; ^3^Department of Life Sciences, Pohang University of Science and Technology, Pohang, South Korea

**Keywords:** protein overexpression, protein purification, *Gracilariopsis chorda*, *Nicotiana benthamiana*, carbonic anhydrase, GcCAα3, enzyme immobilization, CO_2_ hydration

## Abstract

Carbonic anhydrase (CA; EC 4.2.2.1) is a Zn-binding metalloenzyme that catalyzes the reversible hydration of CO_2_. Recently, CAs have gained a great deal of attention as biocatalysts for capturing CO_2_ from industrial flue gases owing to their extremely fast reaction rates and simple reaction mechanism. However, their general application for this purpose requires improvements to stability at high temperature and under *in vitro* conditions, and reductions in production and scale-up costs. In the present study, we developed a strategy for producing GcCAα3, a CA isoform from the red alga *Gracilariopsis chorda*, in *Nicotiana benthamiana*. To achieve high-level expression and facile purification of GcCAα3, we designed various constructs by incorporating various domains such as translation-enhancing M domain, SUMO domain and cellulose-binding domain CBM3. Of these constructs, MC-GcCAα3 that had the M and CBM3 domains was expressed at high levels in *N. benthamiana* via agroinfiltration with a yield of 1.0 g/kg fresh weight. The recombinant protein was targeted to the endoplasmic reticulum (ER) for high-level accumulation in plants. Specific and tight CBM3-mediated binding of recombinant GcCAα3 proteins to microcrystalline cellulose beads served as a means for both protein purification from total plant extracts and protein immobilization to a solid surface for increased stability, facilitating multiple rounds of use in CO_2_ hydration reactions.

## Highlights

- **α** -Carbonic anhydrase GcCA**α** 3 from the red alga *Gracilariopsis chorda* was overexpressed in *Nicotiana benthamiana*, and immobilization on cellulose beads enhanced its stability, facilitating reuse in CO_2_ hydration reactions.

## Introduction

Carbonic anhydrase (CA; EC 4.2.2.1), one of the fastest known enzymes, catalyzes the reversible conversion of carbon dioxide (CO_2_) and water to bicarbonate and protons as follows:

$${\text{CO}}_{2}+{\text{H}}_{2}\text{O}↔{\text{HCO}}_{3}^{-}+{\text{H}}^{+}$$

CAs are Zn-binding metalloenzyme, and their biochemical properties have led to a great deal of interest in their use as biocatalysts for CO_2_ capture from industrial flue gases (Di Fiore et al., 2015). CO_2_ is a major greenhouse gas, and increasing levels of CO_2_ are causing accelerating global warming, which is a major threat to all living organisms worldwide (Pachauri and Reisinger, 2007). Therefore, reducing atmospheric CO_2_ levels is crucial, and various technologies have been developed for CO_2_ capture, including chemical or physical absorption of this gas into a liquid or onto a solid, gas phase separation, and membrane systems (Power et al., 2013). The use of CAs has been proposed for CO_2_ capture (Wang et al., 2011; Di Fiore et al., 2015), and CAs can be used alone or in combination with other technologies to capture CO_2_ from environmental emissions.

Although using CAs as biocatalysts for CO_2_ reduction processes is highly desirable, their efficient use requires many technical advances. First, more thermostable CAs are needed because the temperature of flue gases is generally high. To this end, thermostable CAs have been screened from thermophiles living at high temperatures (Capasso et al., 2012; Luca et al., 2013; Di Fiore et al., 2015). Thermophilic TaCA from *Thermovibrio ammonificans* is stable at 40°C for 75 days and at 60°C for 29 days (Di Fiore et al., 2015). PmCA of *Persephonella marina* is stable at 40°C for 152 days and at 60°C for 75 days (Di Fiore et al., 2015). The thermostability of CAs can be enhanced via mutagenesis. For example, introduction of disulfide bonds can increase the stability of CAs greatly (Jo et al., 2016). In the case of β-carbonic anhydrase DvCA (CA of *Desulfovibrio vulgaris*) mutations that increase interactions between subunits were found to dramatically increase thermostability (Alvizo et al., 2014). Another important characteristic feature required for CAs is long-term stability *in vitro* to allow them to be employed for capturing CO_2_ over a long period of time, and TaCA was shown to possess such properties (Di Fiore et al., 2015). Immobilization of enzymes is another approach for increasing the longevity of enzymes and other proteins (Bhattacharya et al., 2003; Vinoba et al., 2011). Indeed, there have been attempts to immobilize CAs to the surface of SBA15 (Santa Barbara Amorphous-15/Mesoporous Silica/SiO_2_) and polyurethane foam (Vinoba et al., 2011; Migliardini et al., 2014).

Another technical advance required for employing CAs for CO_2_ capture is cost-effective production at a large scale. *Escherichia coli* is the most widely used host to produce CAs and many other enzymes, and recombinant proteins expressed in *E. coli* can often be used without the need for purification. Indeed, CAs have been used together with MDEA (N-Methyldiethanolamine) to capture CO_2_ at high temperatures by adding *E. coli* cells expressing CAs directly to a reactor (Alvizo et al., 2014). Recently, plants have also gained a great deal of interest as a cost-effective production system for recombinant proteins due to advantages such as good biosafety, low investment for growth facilities, and high scalability (Holtz et al., 2015; Tekoah et al., 2015). In addition, plants provide a eukaryotic system that can support post-translational modifications such as N-glycosylation (Schillberg et al., 2003; Holtz et al., 2015). Many different approaches have been employed to produce recombinant proteins at high levels in plants (Sohn et al., 2018; Muthamilselvan et al., 2019). *Nicotiana benthamiana* has been most widely used as a host plant for recombinant protein production due to easy growth, high mass production, and efficient *Agrobacterium* infection for transient expression of heterologous genes (Regnard et al., 2010; Werner et al., 2011; Mortimer et al., 2015). When performing transient expression in *N. benthamiana*, recombinant proteins can be produced within a week after infiltration. Another strategy for producing recombinant proteins in plants is to generate transgenic plants with stable integration of recombinant genes. Once transgenic plants are generated, seeds are used to produce plant biomass at a large scale, thereby simplifying the entire production process for recombinant proteins (Twyman et al., 2002; Holtz et al., 2015; Tekoah et al., 2015; Rosales-Mendoza and Nieto-Gómez, 2018; Park et al., 2019).

In the present study, we investigated whether plants could be used to produce recombinant GcCAα3, an alpha-type CA from the red alga *Gracilariopsis chorda*, at high levels in *N. benthamiana* in a cost-effective manner (Lee et al., 2018). In a previous study, we isolated seven different isoforms of CAs from red algae and found that they are readily expressed in *Arabidopsis* protoplasts (Razzak et al., 2018). We fused various domains to GcCAα3 and tested for high-level expression and accumulation in plant cells, as well as easy purification. A chimeric construct consisting of the binding immunoglobulin protein (BiP) leader sequence, a translational enhancer M domain, GcCAα3, a CBM3 cellulose-binding domain, and an HDEL endoplasmic reticulum (ER) retention motif achieved high-level expression in *N. benthamiana* leaves and easy purification from plant extracts using cellulose beads. We also demonstrated almost irreversible binding of recombinant GcCAα3 proteins to cellulose beads via the CBM3 domain for immobilization of the recombinant proteins to a solid surface for extended stability at high temperature, and multiple rounds of reuse in CO_2_ hydration reactions.

## Materials and Methods

### Plasmid Construction

*CBM3* (AEI55081.1, amino acid [aa] residues 1–159) and *bdSUMO* (residues 21–97) genes were chemically synthesized (Bioneer Corp., Daejeon, South Korea). All synthetic genes were codon-optimized for expression in *N. benthamiana*. We generated chimeric constructs *BiP:M:SUMO:GcCAα3:CBM3:HDEL* (*MSC-GcCAα3*) and *BiP:M:GcCAα3:CBM3:HDEL* (*MC-GcCAα3*) for production of GcCAα3 in *N. benthamiana*. *MSC-GcCAα3* was assembled using the M domain (residues 231–290 of human protein tyrosine phosphatase receptor type C), CBM3, and SUMO through three sequential PCR amplification steps with specific primer sets.

Sequential PCR amplifications were carried out as described below. In the first reaction, PCR product 1 containing the M domain was amplified using *Bam*HI-M domain-F and M domain-*Spe*I-SUMO-R primers. PCR product 2 containing a SUMO domain was amplified using M domain-*Spe*I-SUMO-F and SUMO-MSC-CBM3-R primers. PCR product 3 containing the CBM3 domain was amplified using primers SUMO-MSC-CBM3-F and *Xho*I-CBM3-HDEL-R (Supplementary Table S1). In the second PCR amplification, PCR product 4 was amplified using *Bam*HI-M domain-F and SUMO-MSC-CBM3-R primers with PCR products 1 and 2 as templates. PCR product 5 was amplified using M domain-*Spe*I-SUMO-F and *Xho*I-CBM3_HDEL-R primers with PCR products 2 and 3 as templates (Supplementary Table S1). In the final PCR amplification, PCR products containing M-SUMO-CBM3-HDEL were amplified using *Bam*HI-M domain-F and *Xho*I-CBM3-HDEL-R primers with PCR products 4 and 5 as templates (Supplementary Table S1). After the final round of amplification, PCR products were digested with *Bam*HI and *Xho*I restriction endonucleases, and inserted downstream of BiP in the 326 vector containing a cauliflower mosaic virus (CaMV) 35S promoter with double enhancer (d35S), and a 5′-untranslated enhancer region (5′-UTR):BiP:HSP that was previously digested with *Bam*HI and *Xho*I to give 326-*BiP:M:SUMO:CBM3:HDEL*.

To generate a binary plant expression vector, 326-*BiP:M:SUMO:CBM3:HDEL* was digested with *Xba*I and *Eco*RI restriction endonucleases, and the resulting fragment containing *BiP:M:SUMO:CBM3:HDEL* was ligated into the pCAMBIA1300 binary vector that had been digested with *Xba*I and *Eco*RI restriction endonucleases (Kim et al., 2013). Finally, *GcCA*α*3* was amplified by PCR using *Xma*I-GcCAα3-F and *Kpn*I-GcCAα3-R primers, and inserted downstream of SUMO in the pCAMBIA1300 vector (5′-UTR-BiP-M-SUMO-CBM3-HDEL) using *Xma*I and *Kpn*I restriction endonuclease sites to give *MSC-GcCA*α*3*. To generate *MC-GcCA*α*3*, *GcCA*α*3* was amplified by *Spe*I-GcCAα3-F and *Kpn*I-GcCAα3-R primers (Supplementary Table S1), digested with *Spe*I and *Kpn*I restriction endonucleases, and ligated into *MSC-GcCA*α*3* that was previously digested with *Spe*I and *Kpn*I restriction endonucleases, thereby replacing the *SUMO-GcCA*α*3* fragment. To generate a construct with one additional M domain (*MSC-GcCA*α*3-M*), *M-Linker-CBM3* was PCR amplified using primers *Kpn*I-M domain-F and *Xho*I-CBM3-HDEL-R, and then the PCR product was digested with *Kpn*I and *Xho*I restriction endonucleases, and ligated into *MSC-GcCA*α*3* that had been digested with *Kpn*I and *Xho*I restriction endonucleases.

### Plant Growth

Wild-type *N. benthamiana* plants were grown in a greenhouse under controlled conditions (24°C and 40–65% relative humidity) and a 14 h light/10 h dark cycle with illumination of 140 μmol.m^–2^ s^–1^ for 4–5 weeks.

### Transformation of *Agrobacterium* With Binary Vectors

Plasmid DNA was introduced into *Agrobacterium* by electroporation, and transformed cells were grown on Luria-Bertani (LB) plates supplemented with kanamycin (50 μg/mL) and rifampicin (50 μg/mL) at 28°C for 48 h. A single colony was transferred to 5 mL of LB liquid medium containing kanamycin and rifampicin (50 μg/mL each), and cultured overnight in a shaker. The overnight culture was used to prepare 50 mL cultures for syringe infiltration or 400 mL cultures for vacuum infiltration.

### Agroinfiltration Into Leaf Tissues of *N. benthamiana*

For agroinfiltration into leaf tissues by syringe, 1 mL *Agrobacterium* culture was transferred to 50 mL of LB medium supplemented with kanamycin and rifampicin (50 μg/mL each). After growing for 16 h, cells were collected by centrifugation at 4,500 × *g* for 8 min at 25°C. The supernatant was discarded, and the pellet was resuspended in infiltration buffer (10 mM MES, 10 mM MgSO_4_.7H_2_O, pH 5.7). Finally, the cell suspension was adjusted to an OD_600_ value of 0.8 using infiltration buffer. Acetosyringone was added to the *Agrobacterium* solution to 400 μM final concentration, and the cell suspension was incubated at room temperature for 3 h. Syringe infiltration was carried out using a 1 mL syringe without a needle.

For vacuum infiltration, a 5 mL overnight culture was added to 400 mL of LB medium supplemented with kanamycin and rifampicin (50 μg/mL each). After growing for 16 h, cells were collected by centrifugation at 4,500 × *g* for 8 min at 25°C. The supernatant was discarded, the pellet was resuspended in infiltration buffer (10 mM MES, 10 mM MgSO_4_.7H_2_O, pH 5.7), and the suspension was adjusted to an OD_600_ value of 0.8 by adding infiltration buffer. Plants were placed in a vacuum chamber, and after submerging leaf tissues in *Agrobacterium* suspension, vacuum was applied to 50–400 mbar for 30 or 60 s. Once the vacuum was released, plants were removed from the vacuum chamber and grown for 5–7 days under the same growth conditions used for pre-infiltration growth.

To measure CO_2_ hydration activity of GcCAα3, *MC-GcCA*α*3* was introduced into *Nicotiana benthamiana* plants by agro-infiltration. At 5 DPI, MC-GcCAα3 was purified from transformed plants. Purified proteins were quantified and 10 μg of MC-GcCAα3 were used for CO_2_ hydration activity assay. This whole process was performed three times to get enzymatic activity of GcCAα3.

### Protein Analyses by Sodium Dodecyl Sulfate–Polyacrylamide Gel Electrophoresis and Western Blotting

Total protein extracts were prepared in buffer (50 mM TRIS–HCl (pH 7.5), 150 mM NaCl, 0.1% Triton X-100, 2 mM DTT (dithiothreitol), 1% protease inhibitor cocktail). For SDS-PAGE (sodium dodecyl sulfate–polyacrylamide gel electrophoresis) analysis, protein samples were mixed with 5 × sample buffer (250 mM TRIS–HCl (pH 6.8), 10% SDS, 0.5% Bromophenol Blue, 50% glycerol v/v, and 0.6 M DTT) to a final 1 × concentration and boiled for 5 min. Proteins separated by SDS/PAGE were analyzed by western blotting using anti-CBM3 antibody (Bioapp, Pohang, South Korea). Protein bands were visualized using an enhanced chemiluminescence (ECL) kit (Amersham Pharmacia Biotech), and images were obtained using a LAS 4000 image capture system (Fujifilm, Made in Japan).

### CO_2_ Hydration Activity of GcCAα3 *in vitro*

CO_2_ hydration activity was measured using the Wilbur–Anderson method (Wilbur and Anderson, 1948). Enzyme activity was determined based on the period of time (in seconds) taken for the pH of CO_2_-saturated 20 mM TRIS-sulfate buffer to change from 8.3 to 6.3 at 0°C. Bromothymol blue was used as an indicator of the pH change. At the beginning of reaction, bromothymol blue was added (to give a distinct and visible blue color) to Tris buffer, pH 8.3, showing blue color. After adding CO_2_-saturated water to CA, the CO_2_ hydration reaction rapidly occurs, lowering pH to 6.3 and giving yellow color. The time required for the color change from blue to yellow with or without CA was recorded. The intensity of blue color of bromothymol blue was measures by Spectrophotometer (DU800, UV/VIS, Beckman Coulter) at 470 nm wavelength. When the blue color change to yellow, indicating that reaction was completed, we immediately measured the yellow color intensity by spectrophotometer at 580 nm wavelength. For a blank control, 6.0 mL of ice-cold 20 mM TRIS-sulfate buffer (pH 8.3) was placed into a 20 mL beaker, the temperature was maintained at 0°C, and the pH was recorded. Subsequently, 4 mL of ice-cold CO_2_-saturated water was added to the TRIS-sulfate buffer, and the period of time required for the decrease in pH from 8.3 to 6.3 was determined and denoted as T_*b*_. For enzyme-containing samples, 6.0 mL of ice-cold 20 mM M TRIS-sulfate buffer (pH 8.3) was placed into a 20 mL beaker. The pH was recorded, and freshly diluted enzyme sample (0.1 mL; 10 μg) was added to the beaker, followed by rapid addition of 3.9 mL CO_2_-saturated water, and the period of time required for the decrease in pH from 8.3 to 6.3 was determined and recorded as T_*c*_ (Wilbur and Anderson, 1948; Rickli et al., 1964; Khalifah, 1971; Del Prete et al., 2014). Enzyme activity was then calculated using the following formula:

$$U=10\frac{\left(\frac{T_{b}}{T_{c}}\right)-1}{\mathrm{mg}\mathrm{protein}}.$$

### Thermal Stability Analysis of MC-GcCAα3

Purified microcrystalline cellulose (MCC) bead-bound MC-GcCAα3 was incubated at 70°C in a water bath (Finemould Precision Ind. Co., South Korea). Protein aggregates were removed by centrifugation at 10,000 × *g* for 5 min, and supernatants were stored at 4°C until enzymatic activity was measured. In the case of MCC bead-immobilized proteins, MCC beads with bound MC-GcCAα3 were collected from the incubation solution and used for the CO_2_ hydration reaction. Relative activity (%) was calculated as the ratio of the activity of heat treated enzymes vs. that of untreated enzymes using the following formula:

$$\mathrm{Relative}\mathrm{activity}\left(\%\right)=\frac{\left(\mathrm{Activity}\mathrm{of}\mathrm{heat}\mathrm{treated}\mathrm{enzyme}\right)}{\left(\mathrm{Activity}\mathrm{of}\mathrm{untreated}\mathrm{enzyme}\right)}\times 100.$$

### Production of CaCO_3_ by MC-GcCAα3 via CO_2_ Hydration Reaction

To test the continuous use of MCC bead-bound MC-GcCAα3, a prototype experimental set-up was designed. 10 μg of CA was used in the reaction based on linear graph found after using different amount of CA used for CO_2_ hydration reaction (Supplementary Figure S1). Briefly, MCC beads with bound enzyme (10 μg) were placed in a column, and CO_2_-saturated water was passed through the column with 6 mL/min using a Bio-Rad EP-1 Econo infusion pump (Bio-Rad). 10 mM Ca(OH)_2_ solution was also simultaneously passed through the column with 0.5 mL/min together with CO_2_-saturated water to maintain the pH value to 8 to 9. It is known that CA works with high efficiency at slightly alkaline pH. From the bottom of the column, 100 mL of bicarbonate (HCO_3_^–^) solution was collected in a flask containing 100 mL of 100 mM CaCl_2_ solution (pH 10.5, was adjusted using Tris buffer). Thus, bicarbonate ions reacted immediately with Ca^2+^ ions to produce CaCO_3_. The CA-mediated reaction was continued for 40 days. On each day, CA in the column was continuously used for 8 h. At the final hour of reaction on each day, 100 mL of HCO_3_^–^ solution were collected and mixed with 100 mL of CaCl_2_ to produce CaCO_3_ and precipitates were recovered and dried at 80°C for 30 min. As control, we obtained CaCO_3_ without CA under the same condition. CaCO_3_ formed with CA enzyme and without CA was compared. The amount of CaCO_3_ produced with CA was subtracted from the amount of CaCO_3_ produced with CA to get the CA activity. We carried out this experiment every day, and the relative activity were calculated by comparing the activity of the first day. In graphical representation, we included every 5-day results in Figure 6C.

### Characterization of CaCO_3_ Crystals

The composition of CaCO_3_ precipitates was analyzed using X-ray diffraction (XRD) with Cu Kα radiation (λ = 0.154 nm) on a D/Max-2500/PC instrument (Rigaku). A scanning step of 0.02° and a 2θ range from 20 to 50° were employed. Data were compared with actual XRD data from the Joint Committee on Powder Diffraction Standards (JCPDS). Scanning Electron Microscopy (SEM) was performed on a Mini-SEM SNE 4500 M instrument to determine crystal morphology.

### Statistical Analysis of Experiments

We repeated every experiment at least 3 times to confirm reproducibility. To measure enzymatic activity, we used 10 μg of GcCAα3 prepared from *N. benthamiana*. We prepared GcCAα3 at three biological replicates via three independent transformations (Figure 4A). After confirming the enzymatic activity of 10 μg GcCAα3 at three biological replicates, we prepared ∼1 mg of GcCAα3 and examined the biochemical and biophysical properties of GcCAα3 via *in vitro* experiments at three or five technical replicates (Figures 4B,C, 5A, 6C). For these experiments, we thought that technical replicates are enough to assess statistical significance. We also bought certain amount of human carbonic anhydrase II (hCAII, MERCK, C6624) and used for as a positive control.

## Results

### Design of Chimeric Constructs for Expression of GcCAα3 and Their Transient Expression in *N. benthamiana*

**FIGURE 1:**
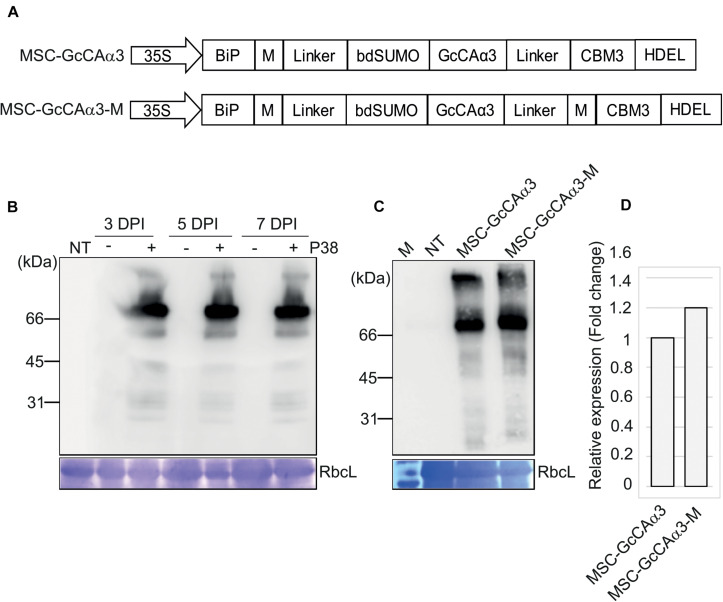
Design of expression constructs for *GcCAα3* and its expression in *Nicotiana benthamiana.* **(A)** Schematic representation of *MSC-GcCAα3* and *MSC-GcCA*α*3-M.* BiP, the leader sequence of BiP; M, the extracellular domain (amino acid residues 231–290) of human protein tyrosine phosphatase receptor type C; linker, GS linker sequence; bdSUMO, the SUMO domain of *Brachypodium distachyon*; CBM3, the CBM domain of *Clostridium thermocellum*; HDEL, ER retention signal; 35S, CaMV 35S promoter. **(B)** Expression of *MSC-GcCAα3* in *N. benthamiana.* Plant leaf tissues were infiltrated with *Agrobacterium* harboring *MSC-GcCAα3* alone (-) or in combination with *Agrobacterium* harboring *P38* (+), and leaf tissues were harvested at 3, 5, and 7 days post-infiltration (DPI). Protein extracts were prepared from leaf tissues and analyzed by western blotting using anti-CBM3 antibody. RbcL, Coomassie Brilliant Blue (CBB)-stained large subunit of the rubisco complex used as a loading control; NT, proteins extracted from non-transformed *N. benthamiana* leaves (negative control). **(C)** Expression of *MSC-GcCA*α*3-M* in *N. benthamiana*. *MSC-GcCA*α*3-M* was expressed as described in panel **(B)**. Protein extracts were analyzed by western blotting using anti-CBM3 antibody. **(D)** Comparison of expression levels of MSC-GcCAα3 and MSC-GcCAα3-M. To quantify expression levels, the band intensity was measured using Multi Gauge V2.2 densitometric software supplied with LAS4000 (Fujifilm, Japan). The expression level of MSC-GcCAα3-M is represented as a value relative to that of MSC-GcCAα3.

In a previous study, we isolated multiple CAs from the red alga *G. chorda*, and found that all were well expressed in *Arabidopsis* protoplasts (Razzak et al., 2018). Herein, we examined whether any of these CAs could be expressed at high levels in plants for the purpose of capturing CO_2_ from flue gases. First, we tested GcCAα3, an alpha-type CA. To express GcCAα3 at high levels, we designed recombinant construct *MSC-GcCA*α*3* for transient expression in *N. benthamiana* after *Agrobacterium*-mediated infiltration. MSC-GcCAα3 consisted of the BiP leader sequence for targeting to the ER, the M domain from human protein tyrosine phosphatase receptor type C (CD45) as a translation-enhancing domain, a SUMO domain to increase solubility of the recombinant protein, GcCAα3, CBM3 of *Clostridium thermocellum* as an affinity tag for purification, and the HDEL motif at the C-terminal end to induce accumulation of recombinant proteins in the ER. In a previous study, the SUMO domain was shown to increase protein solubility in *E. coli* (Jeffrey et al., 2006). CBM3 was also used to immobilize recombinant proteins to the solid surface (Wan et al., 2011). We inserted a linker sequence between domains to give flexibility to the fused domains (Figure 1A).

We examined the protein level of MSC-GcCAα3 in *N. benthamiana* after *Agrobacterium*-mediated infiltration. Plant leaf tissues were infiltrated with *Agrobacterium* harboring *MSC-GcCA*α*3* with or without *Agrobacterium* harboring *P38*, the gene encoding the coat protein of Turnip crinkle virus, as a gene-silencing suppressor (Thomas et al., 2003). Agroinfiltrated leaves were harvested 3, 5, or 7 days post-infiltration (DPI), and total protein extracts from leaf tissue were analyzed by western blotting with anti-CBM3 antibody. A specific band with a molecular weight 70 kDa was detected in leaf tissues infiltrated with Agrobacteria expressing both *MSC-GcCA*α*3* and *P38*. The intensity of the band at 5 and 7 DPI was similar, indicating that expression of MSC-GcCAα3 protein reached a maximum at 5 DPI (Figure 1B). However, in the absence of co-expressed P38, anti-CBM3 antibody did not detect any specific bands, indicating that co-expression of P38 is critical for high-level expression of recombinant genes in *N. benthamiana*. We designed a new construct, *MSC-GcCAα3-M*, containing an additional M domain downstream of *GcCA*α*3* to increase the expression level. *MSC-GcCAα3-M* was introduced into *N. benthamiana* leaf tissues by *Agrobacterium*-mediated infiltration, and its expression level was examined. The expression level of MSC-GcCAα3-M was slightly higher than that of MSC-GcCAα3 (Figures 1C,D), indicating that the additional M domain further enhanced the expression level, albeit only modestly.

**FIGURE 2:**
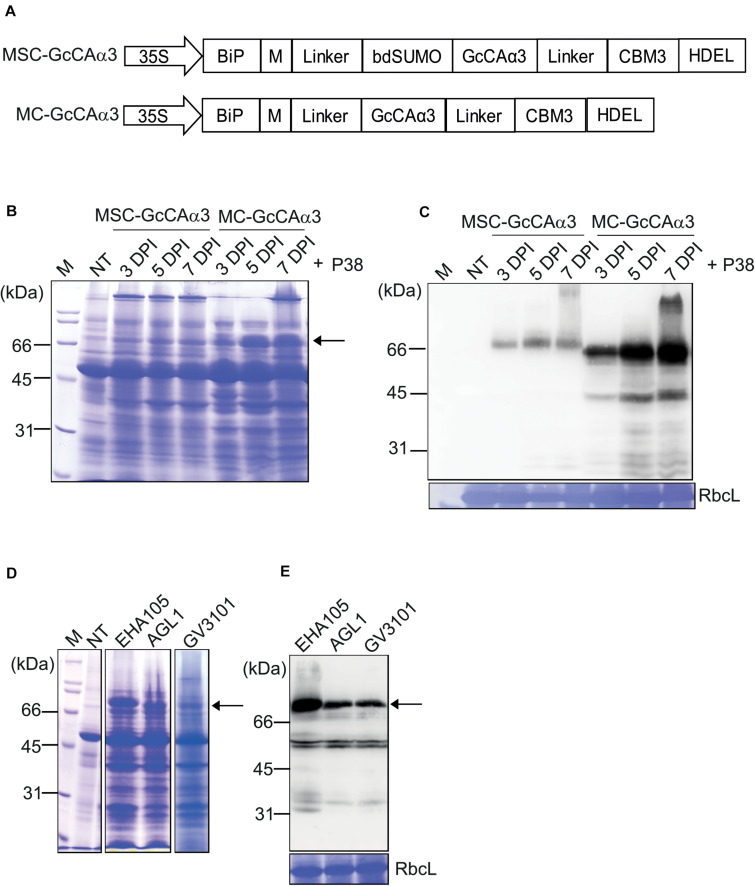
The *MC-GcCA*α*3* construct without a SUMO domain displays high-level expression in *N. benthamiana.* **(A)** Schematic representation of *MSC-GcCA*α*3* and *MC-GcCA*α*3*. **(B,C)** Expression levels of MSC-GcCAα3 and MC-GcCAα3. Leaf tissues of *N. benthamiana* grown for 4–5 weeks were infiltrated with *Agrobacterium* harboring *MSC-GcCAα3* or *MC-GcCAα3.* In addition, *Agrobacterium* harboring *P38* was co-infiltrated. **(B)** Protein extracts were prepared from leaf tissues harvested at the indicated time points and analyzed by SDS-PAGE. After electrophoresis, the gel was stained with CBB. **(C)** After SDS-PAGE, proteins were detected by western blot analysis using anti-CBM3 antibody. RbcL, the large subunit of the rubisco complex stained with CBB used as a loading control; NT, non-transformed control plants; M, molecular weight standard. Expression levels of proteins were quantified based on band intensity of western blots. The signal intensity of bands in panel **(C)** was measured using Multi Gauge V2.2 densitometric software supplied with LAS4000, and values are represented as the fold-change relative to 3 DPI of MSC-GcCAα3. The *MC-GcCA*α*3* is expressed at different levels in *N. benthamiana* transformed with different agrobacterium strains. Three different *Agrobacterium* strains (EHA105, AGL1, and GV3101) harboring *MC-GcCA*α*3* were used to infiltrate leaf tissues of *N. benthamiana* grown for 4–5 weeks. A group co-infiltrated with *Agrobacterium* harboring *P38* was included. Protein extracts (10 μg total soluble proteins) from leaf tissues harvested at 5 DPI were analyzed SDS-PAGE and western blotting using anti-CBM3 antibody. **(D,E)** Comparison of expression levels of MC-GcCAα3 among different *Agrobacterial* strains. Three different *Agrobacterial* strains, EHA105, AGL1 and GV3010, were used to express MC-GcCAα3 in plant tissues. Protein extracts were prepared from leaf tissues at 5 DPI and separated on an SDS-PAGE gel. The gel was stained with CBB **(D)** or analyzed by western blotting using anti-CBM3 antibody **(E)**. The arrows indicate MC-GcCAα3.

In MSC-GcCAα3, we added various domains to *GcCAα3* for the purpose of high-level expression in a soluble form in plants. However, we wondered whether these domains were necessary for expressing GcCAα3 at high levels in plants. Although GcCAα3 originated from a red alga, it was expressed well and in soluble form in *Arabidopsis* protoplasts (Razzak et al., 2018), indicating that the SUMO domain used to increase the solubility of recombinant GcCAα3 protein in *N. benthamiana* may not be necessary. To test this hypothesis, we generated the *MC-GcCA*α*3* construct by deleting the SUMO domain (Figure 2A) and examined expression MC-GcCAα3 in leaf tissues of *N. benthamiana* following *Agrobacterium-*mediated transformation. Again, to increase the expression level, P38 was co-expressed with MC-GcCAα3. After agroinfiltration, total protein extracts were prepared at 3, 5, and 7 DPI, and analyzed by western blotting using anti-CBM3 antibody. As a control, MSC-GcCAα3 was included in the analysis. Anti-CBM3 antibody detected a band at ∼70 kDa, together with minor bands below the main band (Figures 2B,C). However, the main difference was the expression level, which was several fold higher for MC-GcCAα3 than MSC-GcCAα3 (Figure 2C).

The comparision of protein expression level of MSC-GcCAα3 and MC-GcCAα3 were determined based on the band intensity of western blots using the same amounts of total protein loading on SDS-PAGE gels. The amount of MSC-GcCAα3 and MC-GcCAα3 in total protein extracts was quantified by comparing band intensity in the western blot image. The signal intensity of the protein bands were measured using densitometric software. The quantity of MSC-GcCAα3 was represented in an arbitrary unit (A.U.) at a 10 log scale (Log_10_). According to band intensity measurement, the protein expression level of MC-GcCAα3 is approximately 10-fold higher than that of MSC-GcCAα3 (Figure 2C).

Total protein extracts were analyzed by SDS-PAGE followed by Coomassie Brilliant Blue (CBB) staining, and MC-GcCAα3 was one of the most highly expressed protein species detected. In fact, the band intensity was approximately half that of the large subunit of the rubisco complex (Figure 2B). These results indicate that removal of the SUMO domain led to greatly enhanced expression of recombinant *GcCAα3*. However, it is not currently understood how removal of the SUMO domain yielded higher expression of the recombinant protein. The expression level of MC-GcCAα3 increased with time. The increased expression was observed at 5 DPI and reaches highest at 7 DPI. GcCAα3 was a glycoprotein when expressed in plants (Razzak et al., 2018). The *GcCA*α*3* expression construct contained BiP leader sequence at the N-terminus for ER targeting so that carbonic anhydrase can be glycosylated. Moreover, we added HDEL an ER retention signal at the C-terminus to induce accumulation of protein in the ER. Therefore this protein should be targeted to the ER and likely glycosylated in the ER. MC-GcCAα3 expressed in *N. benthamiana* was glycosylated, indicating that it is localized in the ER (Supplementary Figure S2).

Next, we examined whether the expression level of MC-GcCAα3 was influenced by *Agrobacterium* strains. In previous studies, certain *Agrobacterium* strains were found to display different expression levels (Norkunas et al., 2018). Herein, we transformed *MC-GcCA*α*3* into three different *Agrobacterium* strains, GV3101, EHA105, and AGL1, and the transformed Agrobacteria were used to infiltrate into *N. benthamiana* leaves independently. After agroinfiltration into leaf tissues of *N. benthamiana*, plant leaves were harvested at 5 DPI, and total protein extracts were prepared and analyzed by SDS-PAGE followed by western blotting using anti-CBM3 antibody. *Agrobacterium* strain EHA105 achieved the highest level of expression (Figures 2D,E).

### Purification of MC-GcCAα3 From Total Protein Extracts Using MCC Beads

We aimed to purify MC-GcCAα3 from plant extracts, but the cost of purification is an important consideration. In a previous study, to capture CO_2_ in flue gases, recombinant CA expressed in *E. coli* was used without purification (Jo et al., 2013). Thus, purification costs for recombinant GcCα3 proteins from plant extracts should be minimized if possible. To purify recombinant GcCAα3 proteins, we included the CBM3 domain at the C-terminus of GcCAα3 as an affinity tag. CBM3 displayed tight binding to MCC, an abundant and cheap biomaterial (Cazypedia, 2018). MCC beads have been used as an affinity tag for protein purification from extracts of *E. coli*, fungi, and plants (Wan et al., 2011; You and Zhang, 2013; Wang and Hong, 2014; Sohn et al., 2018; Islam et al., 2019).

First, we determined the binding efficiency of CBM3-fused MC-GcCAα3 for MCC. To determine the binding capacity of MCC beads for MC-GcCAα3, different amounts (5–50 μg) of total MC-GcCAα3 proteins expressed in *N. benthamiana* were incubated with 10 mg MCC beads. After binding, MCC beads and supernatants (unbound fractions) were collected separately. MCC beads were washed four times, and proteins bound to beads (bound fractions) were released by boiling in sample buffer. Proteins in bound and unbound fractions were separated by 10% SDS-PAGE and analyzed by western blotting with anti-CBM3 antibody. We found that MC-GcCAα3 in 20 μg (4 μL) total protein extracts was over the saturation level for 10 mg MCC beads, as indicated by the fact that significant amount of MC-GcCAα3 was detected in the unbound fraction (Supplementary Figure S3). A previous study showed that 10 mg MCC beads can bind 20 μg CBM3-conjugated proteins (Islam et al., 2019). These results suggest that a ratio of 10 mg MCC beads to 20 μg total protein extracts may be suitable for purification of MC-GcCAα3 from total protein extracts prepared under the conditions employed herein (Supplementary Figure S3).

**FIGURE 3:**
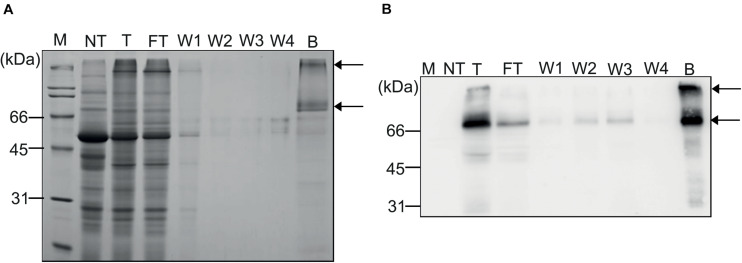
Purification of recombinant MC-GcCAα3 protein from *N. benthamiana* leaf extracts using MCC beads. **(A,B)** Purification of MC-GcCAα3 from plant extracts. Total protein extracts (40 mL) from leaf tissues of *N. benthamiana* were incubated with MCC beads at a 5:1 ratio (v/g). Flow-through fractions were collected and saved for SDS-PAGE and western blot analyses. MCC beads were washed four times with wash buffer, and wash solutions (W1 to W4) were saved for analysis. Proteins bound to MCC beads were eluted by boiling. **(A)** Total protein extracts, FT fractions, wash solutions, and proteins eluted from MCC beads were separated by SDS-PAGE, and gels were stained with CBB. **(B)** After SDS-PAGE, proteins were also analyzed by western blotting using anti-CBM3 antibody. Arrows indicate monomers and oligomers of MC-GcCAα3. M, protein size standard; NT, total protein extract from non-transformed *N. benthamiana*; T, total protein extracts; FT, flow-through fraction; Ws, wash fraction; B, proteins eluted from MCC beads by boiling.

Based on the binding capacity of MCC beads for MC-GcCAα3 (20 μg total protein extracts per 10 mg MCC beads), total protein extracts from *N. benthamiana* leaf tissues were mixed with MCC beads, beads were washed four times, and bound proteins were released by boiling. We collected all flow-through and wash fractions, proteins were eluted from MCC beads by boiling, and all fractions were analyzed by SDS-PAGE followed by western blotting using anti-CBM3 antibody. Also, the SDS-PAGE gel was stained with CBB. Western blotting analysis showed that the major portion of MC-GcCAα3 proteins was detected in the elution fraction at two positions (70 and ∼200 kDa), together with small amounts in the flow-through and wash fractions, indicating that MC-GcCAα3 bound strongly to MCC beads (Figure 3). CBB staining confirmed that most of the plant proteins were present in the flow-through fraction, and only small amounts of proteins were detected in the four wash fractions. The CBB-stained gel showed that the elution fraction contained two major bands, one at 70 kDa and the other at the top of the gel, consistent with western blotting analysis, together with a few minor protein bands below 70 kDa. Minor bands were also detected using anti-CBM3, indicating that they are degradation products of MC-GcCAα3. Thus, purification of MC-GcCAα3 using MCC beads yielded highly purified protein from extracts of *N. benthamiana* leaf tissues.

Next, we quantified the level of MSC-GcCAα3 expressed in *N. benthamiana* and then purified the proteins using MCC beads. To quantify the expressed MSC-GcCAα3, we generated the *Hisx6:GcCAα3:CBM3* construct and expressed it in *E. coli BL21* (*DE3*) cells. Recombinant Hisx6:GcCAα3:CBM3 proteins were purified from *E. coli* extracts using Ni^2+^-NTA affinity column chromatography (Supplementary Figure S4). The concentration of purified Hisx6:GcCAα3:CBM3 was measured by the Bradford method (Bradford, 1976). Different amounts (25–100 ng) of Hisx6:GcCAα3:CBM3 proteins were separated by SDS-PAGE using various volumes (3–45 μg) of total protein extracts containing MSC-GcCAα3 expressed in *N. benthamiana*, and were analyzed by western blotting using anti-CBM3 antibody. By comparing the band intensities, the amount of MSC-GcCAα3 in 3 μg total protein extract was found to be equivalent to 25 ng Hisx6:GcCAα3:CBM3. The volume of total protein extracts was 5 mL for 1 g *N. benthamiana* leaf. Therefore, the expression level of MSC-GcCAα3 was 100 μg/g fresh weight (FW; Supplementary Figure S5A).

The expression level of MC-GcCAα3 was quantified based on western blotting band intensity. Total protein extracts were prepared from *N. benthamiana* leaves infiltrated with *Agrobacterium* harboring *MC-GcCAα3* together with *Agrobacterium* harboring *P38* at a ratio of 10 mL buffer to 1 g leaf tissue. Varying amounts (3–45 μg) of total protein extracts together with Hisx6:GcCAα3:CBM3 expressed in *E. coli BL21* (*DE3*) were analyzed by western blotting using anti-CBM3 antibody (Supplementary Figure S5B). The band intensities of MC-GcCAα3 at 70 and 200 kDa were combined and compared with the intensity of the Hisx6:GcCAα3:CBM3 band. We estimated that the amount of MC-GcCAα3 in 3 μg total protein extracts was comparable to 100 ng Hisx6:GcCAα3:CBM3 (Supplementary Figure S5B). Thus, 10 mL total protein extract contained ∼1,000 μg MC-GcCAα3, indicating that the expression level was 1 mg/g FW, which is approximately 10-fold higher than that of MSC-GcCAα3 (Figure 2C). For subsequent analysis, we used *MC-GcCA*α*3* as the *GcCA*α*3* expression construct.

### MC-GcCAα3 Immobilized on MCC Beads Is More Thermostable in GcCAα3-Catalyzed CO_2_ Hydration Reactions

Reusability and stability *in vitro* are important considerations when using enzymes as biocatalysts for industrial purposes. Previous studies showed that immobilization of proteins on solid surfaces can increase stability and allow multiple rounds of use in reactions (Vinoba et al., 2011). Figure 3 shows that CBM3 bound tightly to MCC beads. To further test the binding of MC-GcCAα3 to MCC beads, we treated MC-GcCAα3-bound MCC beads in various solutions, including 100 mM NaCl, 1 M Na_2_CO_3_ (pH 11.5), 2.1 M MDEA (pH 11.15), 10 mM NaOH (pH 12.0), 0.1 mM HCl (pH 4.0), and 10 mM KOH (pH 12). The amount of protein bound to MCC beads was examined after 24 h of incubation. Proteins remaining bound to MCC beads were eluted by boiling, and proteins present in the incubation media were collected to estimate the degree of protein release from MCC beads. Proteins were analyzed by western blotting using anti-CBM3 antibody. Under all conditions tested, MC-GcCAα3 was not released from MCC beads (Supplementary Figure S6), indicating that MC-GcCAα3 binds tightly to MCC beads. Furthermore, these results suggest that MCC bead-immobilized MC-GcCAα3 can be employed under a wide range of conditions.

**FIGURE 4:**
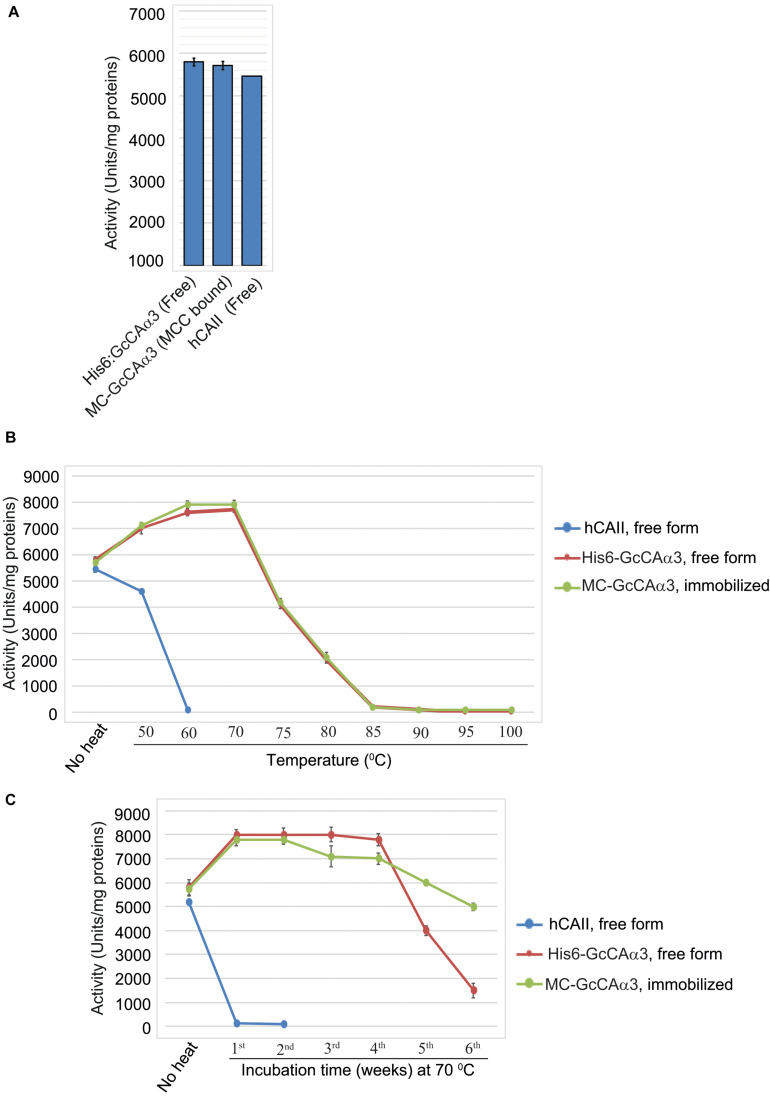
MC-GcCAα3 immobilized on MCC beads is as active as the free form and displays enhanced thermal stability at 70°C. **(A)** Comparison of the CO_2_ hydration activity of free (His_6_:GcCAα3) and MCC bead-bound MC-GcCAα3. His_6_:GcCAα3 was expressed and purified from *E. coli BL21 (DE3)*, and MC-GcCAα3 immobilized on MCC beads (GcCAα3) was purified from Agroinfiltarted *N. benthamiana* leaf tissues. CO_2_ hydration activity was measured by the Wilber–Anderson method and is represented as specific activity. To measure the enzymatic activity of GcCAα3, His_6_:GcCAα3 and MC-GcCAα3 were prepared three times via three independent transformations. Human carbonic anhydrase II (hCAII, MERCK) was used as a positive control. In the case of hCAII which was bought from MERCK, it was considered as a single biological sample. Error bars = standard deviation (*n* = 3). **(B)** Free His_6_:GcCAα3 and MCC bead-bound MC-GcCAα3 both display stability up to 70^0^C. We incubated purified His_6_:GcCAα3 and MCC bead-bound MC-GcCAα3 at different temperature ranging from 50 to 100°C for 10 min, then measured the CO_2_ hydration activity. Thermal stability was determined at three technical replicates. hCAII was used as a positive control. Error bars = standard deviation (*n* = 3). **(C)** Comparison of the long-term thermal stability of free His_6_:GcCAα3 and MCC bead-bound MC-GcCAα3. Enzyme samples were incubated at 70°C for the indicated durations (1–6 weeks). Protein aggregates were removed by centrifugation at 10,000 × *g* for 5 min. CO_2_ hydration activity was measured every week by the Wilber–Anderson method as described in the section “Materials and Methods.” No heat indicates the CO_2_ hydration activity of free GcCAα3 or MCC bead-bound MC-GcCAα3 without heat treatment. One unit corresponds to the CA-catalyzed CO_2_ hydration activity required to reduce the pH of 20 mM TRIS buffer from 8.3 to 6.3 in 1 min at 0°C. For CO_2_ hydration activity, we measured enzymatic activity at 5 technical replicates at each time point. Error bars = standard deviation (*n* = 5).

Next, we examined the CO_2_ hydration activity of MC-GcCAα3 immobilized on MCC beads. To test the effect of immobilization on MC-GcCAα3 activity, we compared the enzymatic activity of immobilized and free forms of GcCAα3 by monitoring the decrease in pH resulting from the GcCAα3-catalyzed CO_2_ hydration reaction. Free His_6_:GcCAα3 was prepared using *E. coli*. Specific activity was calculated using the Wilbur–Anderson formula (Wilbur and Anderson, 1948), and was 5796 Wilber–Anderson units (WAU)/mg for His_6_:GcCAα3 compared with 5711 WAU/mg for the immobilized form (Figure 4A), indicating that the activity was nearly equivalent.

One important advantage of immobilization of enzymes on a solid surface is an increase in stability (Vinoba et al., 2011). We examined whether immobilization of MC-GcCAα3 on MCC beads had any effect on stability. To examine stability, we incubated purified His6:GcCAα3 and MCC bead-bound MC-GcCAα3 at different temperature ranging from 50 to 100^0^C for 10 min, then measured the CO_2_ hydration activity. Free His6:GcCAα3 and MCC bead-bound MC-GcCAα3 both display stability up to 70^0^C whereas human carbonic anhydrase II (hCAII, MERCK, C6624) shows stability up to 50°C (Figure 4B).

Furthermore, we incubated His_6_:GcCAα3 (free form) and MCC bead-bound MC-GcCAα3 at 70°C for up to 42 days, and then measured the GcCAα3-catalyzed CO_2_ hydration activity *in vitro*. Incubation of MC-GcCAα3 at 70°C for 1 day led to an increase in CO_2_ hydration activity of 38%. The hydration activity was then maintained at this elevated level during 4 weeks of heating at 70°C for both free and immobilized enzyme, although MCC bead-immobilized MC-GcCAα3 showed a slight decrease in activity during the third and fourth weeks. However, free His_6_:GcCAα3 exhibited a rapid drop in hydration activity after 5 and 6 weeks, whereas MCC bead-immobilized MC-GcCAα3 displayed a slower decrease in CO_2_ hydration activity at these time points, indicating that immobilization of MC-GcCAα3 on MCC beads enhanced the stability of MC-GcCAα3 (Figure 4C).

### Immobilized MC-GcCAα3 Can Be Reused for Multiple Rounds in CO_2_ Hydration Reactions *in vitro*

**FIGURE 5:**
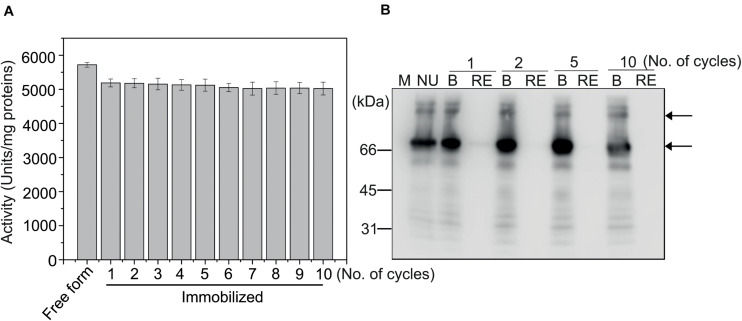
MC-GcCAα3 immobilized on MCC beads can be used in multiple rounds of CO_2_ hydration. **(A)** CO_2_ hydration activity of MC-GcCAα3 immobilized on MCC beads. Enzymatic activity was measured using the Wilber–Anderson method. MC-GcCAα3 (10 μg) bound to MCC beads was added to 20 mM TRIS buffer (pH 8.3), and the time required to change the pH from 8.3 to 6.3 was measured after adding CO_2_-saturated water. After reaction, MCC bead-bound MC-GcCAα3 was recovered and used again for multiple CO_2_ hydration reactions (as indicated by the number of cycles). The enzymatic activity was measured at 5 technical replicates. Free His_6_:GcCAα3 served as a control. The CO_2_ hydration activity of GcCAα3 is presented in units/mg enzyme. Error bars = standard deviation (*n* = 5). **(B)** MC-GcCAα3 is not lost from MCC beads during CO_2_ hydration reaction. After CO_2_ hydration reaction for the indicated number of cycles, MC-GcCAα3 recovered from MCC beads by boiling **(B)** and protein in the CO_2_ hydration solution (RE) were analyzed by western blotting using anti-CBM3 antibody. NU, unused enzyme; M, protein size standards.

We examined whether immobilized MC-GcCAα3 could be reused for multiple rounds of catalysis. After the CO_2_ hydration reaction, MCC beads containing bound MC-GcCAα3 were collected and used again in the CO_2_ hydration reaction, and this was repeated up to ten times. Even at the tenth repeat, the CO_2_ hydration activity of MC-GcCAα3 was >95% that of the first reaction (Figure 5A), indicating that MC-GcCAα3 could be reused for multiple rounds of catalysis without losing activity. To confirm that MC-GcCAα3 remained bound to MCC beads, MC-GcCAα3 was released from MCC beads by boiling after the first, third, fifth, and tenth reactions, and was analyzed by western blotting using anti-CBM3 antibody. The same amount of MC-GcCAα3 was released from MCC beads after the first and tenth reactions, and no protein was detected in the reaction solution (RE), confirming that proteins remained bound to MCC beads (Figure 5B).

**FIGURE 6:**
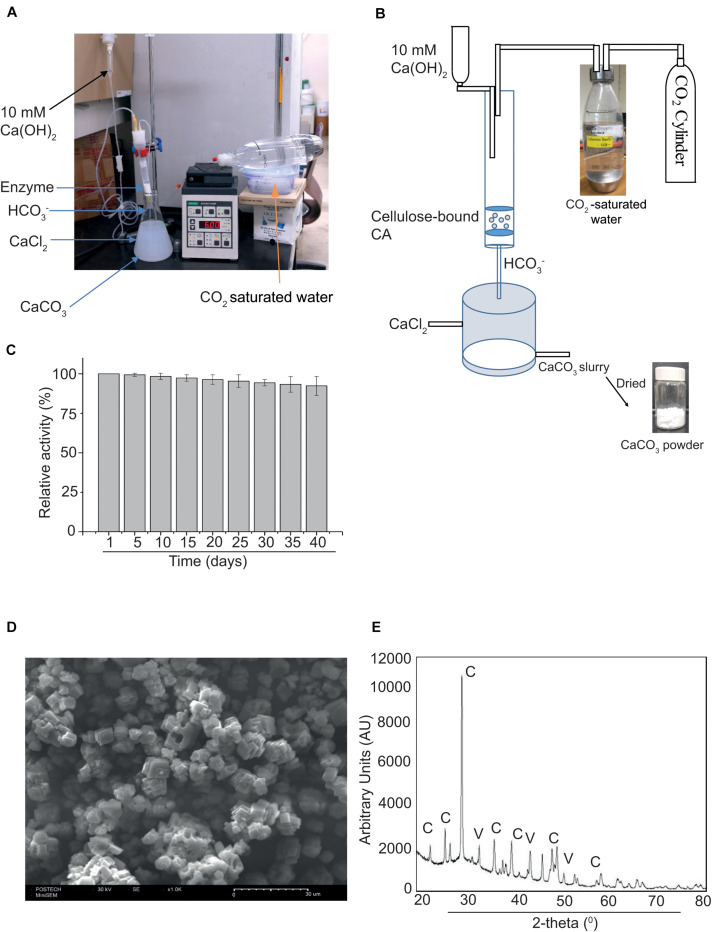
MC-GcCAα3 immobilized on MCC beads retains 90% CO_2_ hydration activity for 40 days. **(A)** A prototype reactor set up for measuring the long-term activity of MCC bead-bound MC-GcCAα3. **(B)** The schematic diagram of the reactor to continuously produce CaCO_3_ through CO_2_ hydration by MCC bead-bound MC-GcCAα3. CO_2_-saturated water was prepared by bubbling CO_2_ in water. CO_2_-saturated water was pumped into the column. **(C)** Long-term activity of MCC bead-bound MC-GcCAα3. 10 μg of MCC bead-bound MC-GcCAα3 was placed in a column, and freshly prepared CO_2_-saturated water was continuously pumped into the column at a flow rate of 6 mL/min using an infusion pump. 10 mM Ca(OH)_2_ was also poured into the column at a rate of 0.5 mL/min to keep the pH of CO_2_-saturated water in the range of pH 8 to 9 for higher GcCAα3 activity. The GcCAα3-mediated CO_2_ hydration reaction was continuously run for 8 h in a day and at final hour of the day, 100 mL bicarbonate solution was mixed with 100 mL of 100 mM CaCl_2_ (pH 10.5) in a beaker. CaCO_3_ precipitates were collected and dried at 80°C for 30 min. MCC beads without enzyme were used as control. To calculate enzyme activity, the amount of CaCO_3_ powder produced without GcCAα3 was subtracted from the amount of CaCO_3_ powder produced with GcCAα3. Enzyme activity was measured everyday. However, in the graphical representation, we included data of 5-day intervals for 40 days. Enzymatic activity on the first day was considered 100%, and enzymatic activity at other time points are represented relative to this initial activity. The enzymatic activity was measured at 3 technical replicates. Error bars = standard deviation (*n* = 3). **(D)** Morphology of precipitated CaCO_3_ determined by scanning electron microscopy (SEM). Scale bar = 30 μm. **(E)** Structural analysis of CaCO_3_ by X-ray diffraction (XRD). AU, arbitrary units; C, calcites; V, vaterites.

Finally, we tested the use of MCC bead-bound MC-GcCAα3 in the production of CaCO_3_ from CO_2_ via a GcCAα3-catalyzed CO_2_ hydration reaction. A prototype experimental system was set up in such a way that MCC beads containing bound MC-GcCAα3 were placed in a column, and CO_2_-saturated water was passed through the column by an infusion pump. Bicarbonate (HCO_3_^–^) ions resulting from the GcCAα3-catalyzed CO_2_ hydration reaction were eluted from the column and immediately mixed with CaCl_2_ solution (pH 10.5) in a beaker placed under the column (Figures 6A,B). In this way, bicarbonate ions immediately reacted with Ca^2+^ ions, thereby producing CaCO_3_. CaCO_3_ precipitates were recovered from solution and dried at 60°C for 4 h. The production of CaCO_3_ was examined for 40 days using the same preparation of MCC bead-bound MC-GcCAα3. Enzyme activity was calculated based on the production of CaCO_3_. After 40 days, the amount of CaCO_3_ produced was only 10% less than that after 1 day (Figure 6C), indicating that MC-GcCAα3 retained 90% activity under the conditions employed continuously for 40 days.

The morphology of CaCO_3_ crystals was analyzed by SEM. CaCO_3_ crystals were largely cubic, indicating the presence of calcites (Figure 6D). To further examine the nature of the crystals, we analyzed CaCO_3_ powder by XRD. CaCO_3_ produced an XRD pattern that was identical to that of the authentic standard (Figure 6E), confirming that the powder consisted of CaCO_3_ crystals. Based on the SEM and XRD data, calcite and vaterite were formed at a ratio of 85:15 mol%.

## Discussion

CAs have recently attracted a great deal of attention for their possible use in capturing CO_2_ from industrial flue gases (Di Fiore et al., 2015). Many current technologies for CO_2_ capture from flue gas rely on amine-based chemical catalysts such as MDEA (Rochelle, 2009). Thus, the use of a biocatalyst such as CAs would be highly advantageous, since CA-based CO_2_ absorption is environmentally safer than chemical-based methods. Moreover, CAs can be used together with MDEA to greatly facilitate both CO_2_ absorption from flue gas and CO_2_ release from MDEA solution (Alvizo et al., 2014). For this application, CAs should be produced at a large scale in a cost-effective manner. In the present study, we explored the possibility of using plants as a host system for the large-scale production of CAs, using the red alga isoform GcCAα3 as a model CA for use as a biocatalyst to catalyze CO_2_ absorption.

Plants have been proposed as an ideal system for production of recombinant proteins at a large scale due to the ease and low cost of plant growth, and the low cost of investment for facilities and their maintenance (Regnard et al., 2010; Werner et al., 2011; Mortimer et al., 2015). However, low expression levels of proteins in plants can be an issue for recombinant protein production. Recently, many gene expression vectors have been developed for high-level expression of foreign genes in plants. In general, high-level expression of recombinant proteins in plants can be achieved using RNA virus-based expression vectors such as MagnICON, pEff, and pTRBO (Gleba et al., 2005, 2014; Lindbo, 2007; Mardanova et al., 2017; Muthamilselvan et al., 2019). These vectors are used for transient expression via *Agrobacterium*-mediated infiltration, the fastest method for production of recombinant proteins in plants (Gleba et al., 2005, 2014; Lindbo, 2007; Mardanova et al., 2017). Using these vectors, recombinant proteins can be produced at yields of 1 − 5 g/kg FW. However, expression levels of recombinant proteins in plants can vary greatly for different proteins.

Herein, we designed a non-viral vector-based recombinant construct for high-level expression of GcCAα3 using various functional domains to increase expression level and solubility. With a view to engineering GcCAα3 for industrial application, we fused domains from other proteins to GcCAα3 in the hope that they would prove advantageous without affecting enzymatic activity. We generated a few different recombinant constructs to test the expression of GcCAα3. The M domain is known to increase the expression level of some fusion proteins (Kang et al., 2018), while the SUMO domain can enhance protein solubility (Jeffrey et al., 2006). Thus, we included these two domains to increase expression level and solubility, and the resulting MSC-GcCAα3 was expressed at high yield (100 mg/kg FW). We also generated a construct with two M domains, *MSC-GcCAα3-M*, but expression levels were only slightly higher than for MSC-GcCAα3, indicating that M domain-mediated enhancement of expression was already saturated with a single M domain. However, MC-GcCAα3 not harboring a SUMO domain displayed almost 10-fold higher expression than MSC-GcCAα3. GcCAα3 is a soluble algal protein expressed in plant protoplasts (Razzak et al., 2018), indicating that a domain to increase solubility is not necessary. In fact, CAs localized in chloroplasts are among the most abundant proteins in plants (Fabre et al., 2007), implying that CAs are intrinsically highly soluble proteins. We also found that the *Agrobacterium* strain can affect expression levels when transient expression via *Agrobacterium*-mediated infiltration was used to produce recombinant proteins. By testing multiple chimeric constructs for the expression of *GcCA*α*3*, we achieved a production yield of 1.0 g/Kg FW in *N. benthamiana* leaves expressing MC-GcCAα3 via *Agrobacterium*-mediated infiltration. This expression level is comparable to that achieved by RNA virus-based vectors (Gleba et al., 2005, 2014; Lindbo, 2007; Mardanova et al., 2017). In the case of *N. benthamiana*, a single plant producing 60 g leaf tissue can produce 60 mg MC-GcCAα3 in 5 days after *Agrobacterium*-mediated infiltration.

For large-scale production of GcCAα3, in the future study we will use MC-GcCAα3 to generate transgenic plants. Plant species such as *Nicotiana benthamiana* and *Nicotiana tabacum* that can be easily transformed and give high biomass would be a good choice. After screening elite homolines with high expression of MC-GcCAα3, seeds are used to produce plant biomass at a large scale, thereby simplifying the entire production process for recombinant proteins. For the large scale production of carbonic anhydrase the seeds of transgenic plants will be used to grow large amount of plants.

In addition to high-level expression, another important consideration in reducing the cost of enzyme production is to lower the purification cost. In fact, for production of recombinant proteins, the most expensive step is protein purification from total cell extracts (Schillberg et al., 2019). When recombinant proteins are used for industrial application, protein purity may not be a crucial factor, in contrast to biomedical application. Indeed, a previous study showed that an *E. coli* culture expressing CAs could be used directly for CO_2_ absorption and CO_2_ release from MDEA solution without purification (Alvizo et al., 2014). Similarly, CAs expressed on the surface of *E. coli* can be used to capture CO_2_ without purification (Jo et al., 2013). Thus, the direct use of bacteria expressing CAs is a cost-effective way to use CAs for capturing CO_2_ without any cost incurred from purification. However, using CAs without purification may limit their applications. In the case of CAs produced in plants, plant extracts may not be suitable for direct use in CO_2_ capture from flue gases. In the present study, we focused on cost-effective purification of CAs produced in plants. As an affinity tag for GcCAα3 purification, we included the CBM3 domain, which has a high affinity for cellulose, a cheap and abundant biomaterial (Wan et al., 2011; Cazypedia, 2018; Islam et al., 2019). Moreover, no special conditions were required for binding MC-GcCAα3 to MCC beads, and the binding capacity of MCC beads for MC-GcCAα3 was high (2 − 4 mg/g). The purity of MC-GcCAα3 was sufficient for use in capturing CO_2_ from industrial sources. These results suggest that a large amount of MC-GcCAα3 can be purified with a high degree of purity in a cost-effective manner using our approach.

A previous study showed that the CBM3 domain binds almost irreversibly to MCC beads (Islam et al., 2019). Consistent with this finding, MC-GcCAα3 bound to MCC beads was almost impossible to release under certain conditions such as high and low pH, high salt, and in the presence of MDEA, and boiling of beads was therefore needed. Because CBM3 binds almost irreversibly to MCC beads, CBM3-mediated binding of MC-GcCAα3 to MCC beads during protein purification can also be used as a means to immobilize MC-GcCAα3 onto a solid surface. In fact, there have been many attempts to immobilize CAs on solid surfaces such as SBA15, polyurethane foam, and magnetic polymer (Vinoba et al., 2011; Migliardini et al., 2014; Jing et al., 2015). Immobilization of enzymes to solid surfaces can increase their stability and allow reuse in multiple cycles. Indeed, MC-GcCAα3 immobilized on MCC beads displayed enhanced thermal stability, and the enzyme could be successfully reused for multiple rounds of CaCO_3_ production via the GcCAα3-mediated CO_2_ hydration reaction.

In summary, we demonstrated that GcCAα3 can be produced as a chimeric recombinant protein, MC-GcCAα3, at high yield (1.0 g/Kg FW) in *N. benthamiana* plant tissues. Recombinant MC-GcCAα3 was purified using MCC beads in a cost-effective manner. Moreover, MCC bead-bound MC-GcCAα3 was stable at 70°C for more than 5 weeks and could be used for multiple cycles of CO_2_ hydration reactions to produce CaCO_3_.

## Data Availability Statement

All datasets presented in this study are included in the article/Supplementary Material.

## Author Contributions

MR, JL, and IH conceived the project, MR and IH designed the research and interpreted the results and wrote the manuscript. MR performed the most of the experiments. DL prepared the MSC-GcCAα3 construct. All authors contributed to the article and approved the submitted version.

## Conflict of Interest

The authors declare that the research was conducted in the absence of any commercial or financial relationships that could be construed as a potential conflict of interest.

## Supplementary Material

Supplementary Figure 1 The activity of *MC-GcCAα3* based on pH change of TRIS buffer 8.3 to 6.3 with different amount of *MC-GcCAα3* enzyme used.

Supplementary Figure 2 *GcCAα3* expressed in the ER of *N. benthamiana* leaf cells was N-glycosylated.

Supplementary Figure 3 Binding capacity of microcrystalline cellulose (MCC) beads for CBM3 fusion proteins.

Supplementary Figure 4 Expression and purification of Hisx6:GcCAα3 from *E. coli*.

Supplementary Figure 5 Quantification of *MSC-GcCAα3* and *MC-GcCAα3* produced in *N. benthamiana.*

Supplementary Figure 6 MC-GcCAα3 binds tightly to MCC beads under various conditions and different pH values.

Supplementary Table 1 Primer sequences used to generate the constructs in this study.
