# Supplementary material for: Overexpression and Purification of Gracilariopsis chorda Carbonic Anhydrase (GcCAα3) in Nicotiana benthamiana, and Its Immobilization and Use in CO2 Hydration Reactions
Source: Front Plant Sci. 2020 Nov 19;11:563721. doi: 10.3389/fpls.2020.563721 (PMC7717956; doi:10.3389/fpls.2020.563721)
Supplement: Supplementary Figure 1 — The activity of MC-GcCAα3 based on pH change of TRIS buffer 8.3 to 6.3 with different amount of MC-GcCAα3 enzyme used. [file Data_Sheet_1.docx]

**Overexpression and purification of *Gracilariopsis chorda* carbonic anhydrase (GcCAα3) in *Nicotiana benthamiana*, and its immobilization and use in CO_2_ hydration reactions**

Md Abdur Razzak^1^, Dong Wook Lee^1,2^, Junho Lee^1^ and Inhwan Hwang^1,3^*

^1^Division of Integrative Biosciences and Biotechnology, Pohang University of Science and Technology, Pohang, 37673, Korea.

^2^Department of Bioenergy Science and Technology, Chonnam National University, Gwangju, 61186, South Korea.

^3^Department of Life Sciences, Pohang University of Science and Technology, Pohang, 37673, Korea.

**Highlights: α**-Carbonic anhydrase GcCA**α**3 from the red alga *Gracilariopsis chorda* was overexpressed in *Nicotiana benthamiana*, and immobilization on cellulose beads enhanced its stability, facilitating reuse in CO_2_ hydration reactions.

^*^Corresponding author: To whom correspondence should be addressed

E-mail: [ihhwang@postech.ac.kr](mailto:ihhwang@postech.ac.kr)

Tel: 82-54-279-2128

Fax: 82-54-279-8159

**ORCID ID: 0000-0002-1388-1367 (I.H.)**

**Supplementary Figures**

**
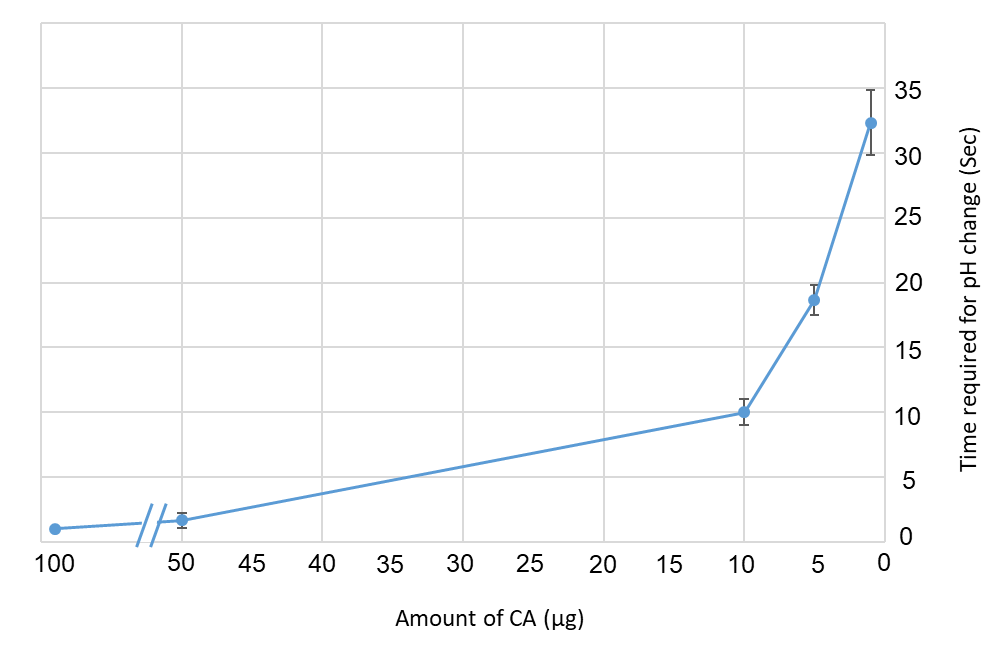
**

**Supplementary Figure S1. The activity of *MC-GcCAα3* based on pH change of TRIS buffer 8.3 to 6.3 with different amounts of *MC-GcCAα3*.**

To optimize the CO_2_ hydration reaction of *MC-GcCAα3*, we used 1, 5, 10, 50 and 100 μg of proteins to test the activity. As expected, we found the activity showed a linear range. The activity of 1, 5, 10 μg of enzyme showed a linear increase. In addition, to be more precise, the enzyme activity was saturated at 50 and 100 μg. The less time required for pH change indicates the faster reaction. Based on these observations, we used 10 μg of *MC-GcCAα3* to test activity of *MC-GcCAα3* in all other experiments.

**
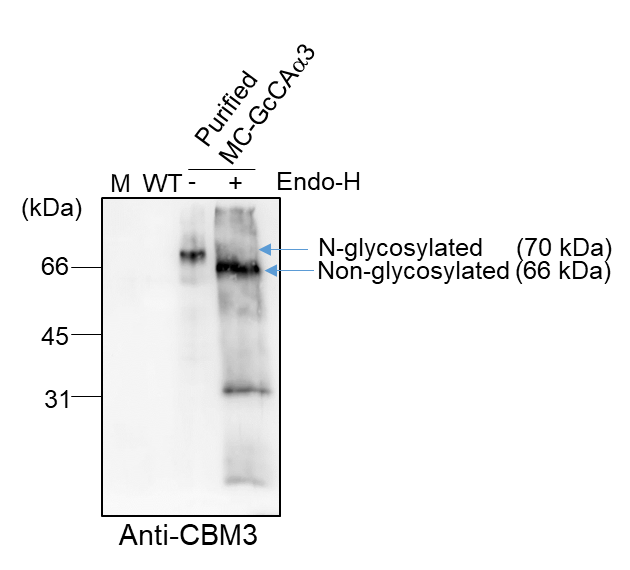
**

**Supplementary Figure S2. GcCAα3** **expressed in ER of *N. benthamiana* leaf cells and glycosylated.**

GcCAα3 were purified from leaf tissues of *N. benthamiana* by MCC. Then the purified proteins were treated with Endoglycosidase-H (Endo-H) for 3 hours at 37°C. Endo-H can deglycosylates the ER protein. Therefore, after treated with Endo-H protein bands appears with reduced size. WT, protein extracts from non-transformed control wild type plants; M, molecular weight standard. (-), untreated and (+), treated protein with Endo-H.

**
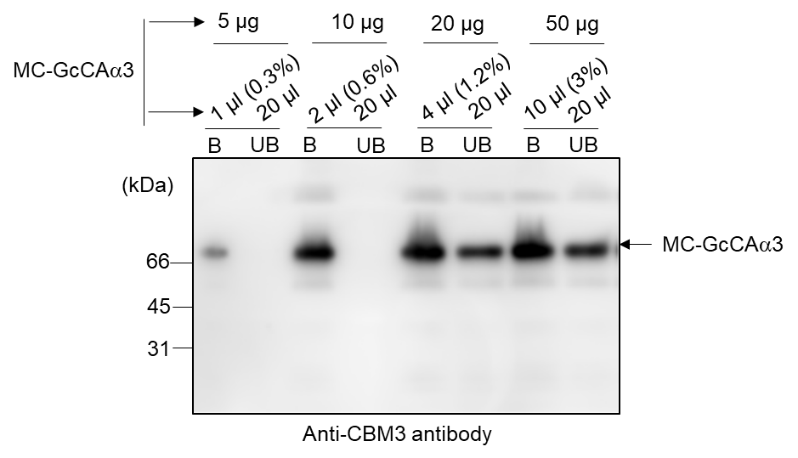
**

**Supplementary Figure S3. Binding capacity of microcrystalline cellulose (MCC) beads for CBM3 fusion proteins.**

To determine the binding capacity of MCC beads for MC-GcCAα3, different amounts (5–50 µg) of total protein extracts from leaf tissues of *N. benthamiana* harvested at 5 DPI after infiltration with a mixture (1:1 ratio) of *Agrobacterium* harboring *MC-GcCAα3* and *Agrobacterium* harboring *P38* were incubated with 10 mg MCC beads in 1 mL buffer. Total protein extracts were prepared at a ratio of 2 mL buffer to 1 g leaf tissue. After binding, MCC beads and supernatant (unbound fraction) were collected separately. MCC beads harboring bound proteins were washed four times with 40 mM TRIS-HCl, pH 7.5. Proteins bound to beads (B fraction) were released by boiling in 300 μL sample buffer (250 mM TRIS-HCl (pH 6.8), 10% SDS, 0.5% Bromophenol Blue, 50% glycerol v/v, 0.6 M DTT). Proteins in 10 µL (3% of the total volume) of the bound fraction or 20 µL (5% of the total volume) of the unbound (UB) fraction were separated by 10% SDS-PAGE and analyzed by western blotting with anti-CBM3 antibody.


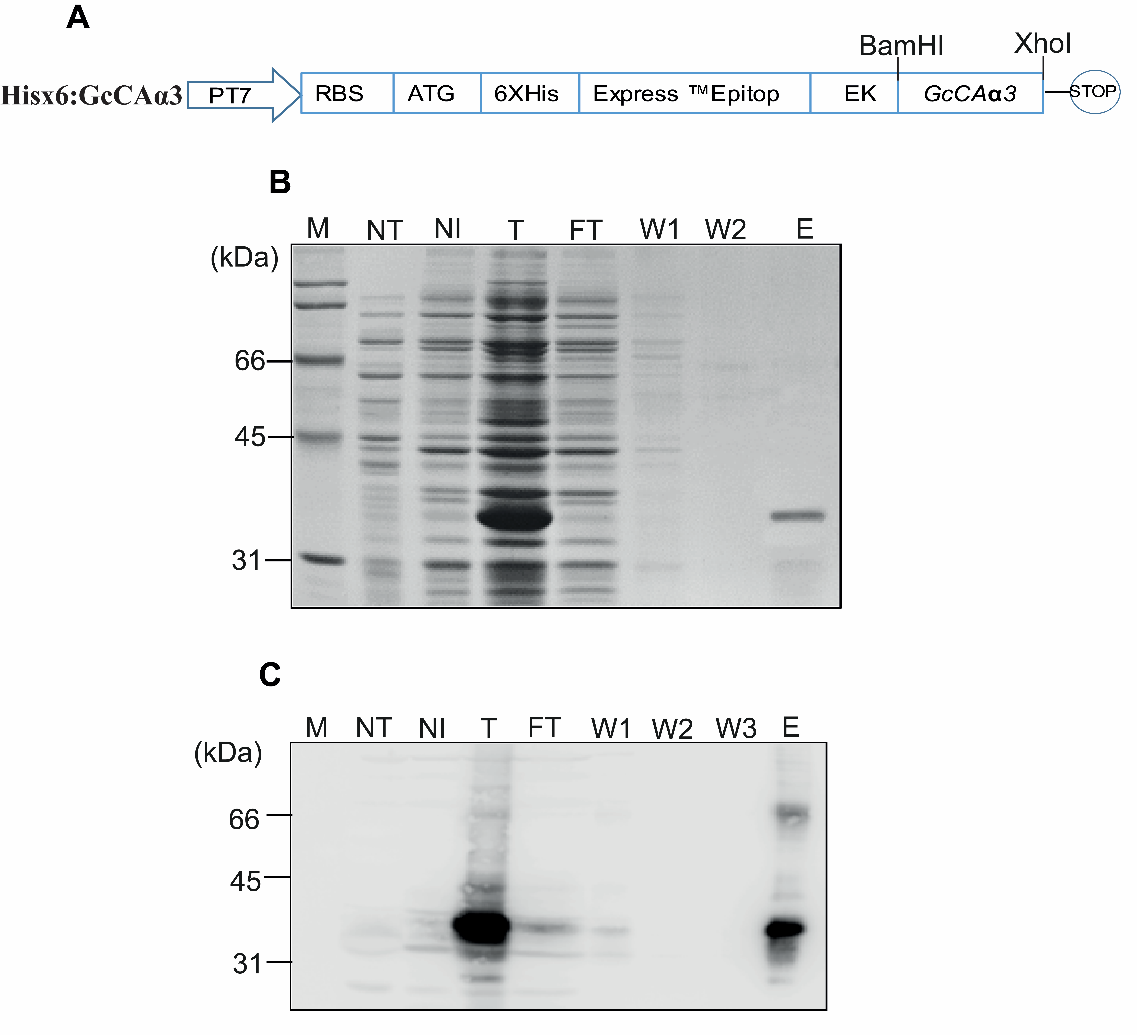


**Supplementary Figure S4. Expression and purification of Hisx6:GcCAα3 from *E. coli*.**

**(A)** Schematic diagram of the *Hisx6:GcCAα3* construct for expression in *E. coli.*

**(B, C)** Expression of His_6_-tagged GcCAα3 in *E. coli*. The construct was introduced into *E. coli* strain BL21 (DE3). *E. coli* cells were cultured in LB medium and expression of Hisx6:GcCAα3 was induced with IPTG for 3 h at 37°C. Cell extracts were prepared from *E. coli* cultures and Hisx6:GcCAα3 was purified using Ni^+^-NTA affinity column chromatography. Purified Hisx6:GcCAα3, total extracts, flow-through, and wash fractions were separated by SDS-PAGE and stained with Coomassie Brilliant Blue (**B**) or analyzed by western blotting using anti-His antibody (**C**). M, molecular weight markers; T, total soluble proteins extracted from *E. coli* after induction with IPTG; NT, total extracts from non-transformed *E. coli*; NI, total extracts from non-induced *E. coli*; FT, flow-through fraction; W, wash fraction; E, eluted fraction. The amount of purified Hisx_6_:GcCAα3 was measured by the Bradford method.

**
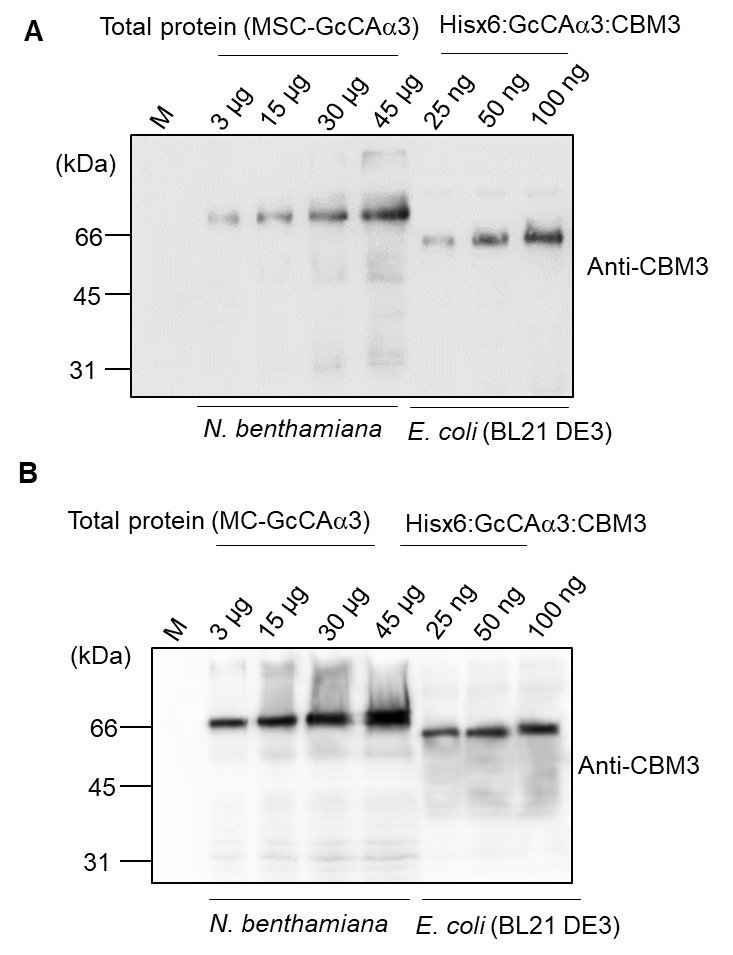
**

**Supplementary Figure S5. Quantification of MSC-GcCAα3 and MC-GcCAα3** **produced in *N. benthamiana.***

(**A**) Quantification of MSC-GcCAα3 in total protein extracts. To prepare total protein extracts, 1 g leaf tissue from *N. benthamiana* infiltrated with a mixture of *Agrobacterium* harboring *MSC-GcCAα*3 and *Agrobacterium* harboring *P38* was harvested at 5 DPI. After grinding in liquid nitrogen, it was homogenized in 5 mL extraction buffer. Total proteins contained in the indicated amounts (3–45 μg) were separated by SDS-PAGE and analyzed by western blotting using anti-CBM3 antibody. As control for quantification, the indicated amounts (25–100 ng) of Hisx6:GcCAα3:CBM3 purified from *E. coli* were loaded on the same gel. The amount of *MSC-GcCAα3* in total protein extracts was quantified by comparing band intensity in the western blot image. The signal intensity of the protein bands was measured using Multi Gauge V2.2 densitometric software (Fujifilm). The quantity of *MSC-GcCAα3* was represented in arbitrary units (A.U.) at a 10 log scale (Log_10_). *MSC-GcCAα3* in 3 μg total protein extracts appears to be equal to 25 ng Hisx6:GcCAα3:CBM3. Quantification indicates that the yield of *MSC-GcCAα3* in *N. benthamiana* was approximately 100 μg/g FW.

(**B**) Quantification of *MC-GcCAα3* abundance in total protein extracts. To quantify the expression level of *MC-GcCAα3*, total protein extracts were prepared from leaf tissues of *N. benthamiana* at 5 DPI after infiltration of a mixture of *Agrobacterium* harboring *MC-GcCAα3* and *Agrobacterium* harboring *P38*. 1 g sample of leaf tissue was ground in liquid nitrogen and homogenized in 10 mL extraction buffer. Due to high expression of *MC-GcCAα3*, the weight of fresh leaf tissues to extraction buffer ratio was 1:10 (1 g leaf tissue: 10 mL extraction buffer). Finally, the indicated amounts (3–45 μg) of total protein extracts were separated by SDS-PAGE. As control for quantification, the indicated amounts (25–100 ng) of purified Hisx6:GcCAα3:CBM3 from *E. coli* were loaded on the same gel. After western blot analysis using anti-CBM3 antibody, the band intensity of *MC-GcCAα3* was quantified by Multi Gauge V2.2 densitometric software supplied with LAS4000, and the amount of protein was estimated by comparing with that of Hisx6:GcCAα3:CBM3. *MC-GcCAα3* in 3 μg total protein extracts appears to be equal to 100 ng Hisx6:GcCAα3:CBM3. The amount of *MC-GcCAα3* was estimated to be 1.0 mg/g FW. M, molecular weight standards.


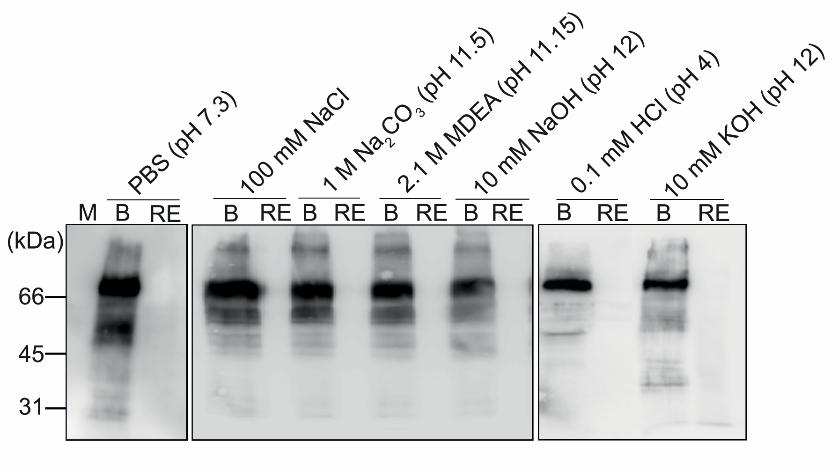


**Supplementary Figure S6. MC-GcCAα3 binds tightly to MCC beads under various conditions and different pH values.**

To test the binding stability of MC-GcCAα3 to MCC beads, MC-GcCAα3-bound MCC beads were incubated in the indicated solutions for 24 h, after which proteins spontaneously released from MCC beads during incubation were collected from the incubation solution (RE), and proteins still bound to MCC beads (B) were eluted by boiling. These fractions were analyzed by western blotting using anti-CBM3 antibody.

**Supplementary Table S1. Primer sequences used to generate the constructs in this study.**

| **Primer names** | **Sequences** |
| --- | --- |
| BamHI-M domain-F | CG GGATCC CG ATGGCAAACATCACTGTGG |
| M domain-SpeI-SUMO-F | CTGCTCCTGATGGTGGAGGAGGTTCTGGTGGTGGATCAACTAGT CATATTAATTTGAAAGTGAA |
| M domain-SpeI-SUMO-R | TTCACTTTCAAATTAATATGACTAGTTGATCCACCACCAGAACCTCCTCCACCATCAGGAGCAG |
| SUMO-MSC-CBM3-F | CAGACTGGTGCCGGCGGT CCCGGGGGTGGAGGAGGTTCA  GGTGGTACC GTATCAGGTAACCTTAAGG |
| SUMO-MSC-CBM3-R | CCTTAAGGTTACCTGATACGGTACCACCTGAACCTCCTCCACCCCCGGGACCGCCGGCACCAGTCTG |
| XhoI-CBM3_HDEL-R | CCGCTCGAGTTAAAGCTCGTCATGACCAGGTTCCTTTCCCCA |
| XmaI_GcCAα3-F | TCCC CCCGGG AACGAGGAAATCGAGGTC |
| KpnI_GcCAα3-R | CGG GGTACC GGACACGTAGCAAGTGAC |
| SpeI_GcCAα3-F | GGACTAGTATGAACTTCTTCGCTACC |
| BamHI_GcCAα3-F | CGGGATCCGAGGAAATCGAGGTCGAC |
| XhoI_GcCAα3-R | CCGCTCGAGTTAGGACACGTAGCAAGT |
| KpnI-M domain-F | CGG GGTACC ATGGCAAACATCACTGTGG |
| XhoI-CBM3_HDEL-R | CCG CTCGAG TTAAAGCTCGTCATGACCAGGTTCCTTTC  CCCA |
